# The effect of velocity-based resistance training (VBT) on lower-limb strength performance in male collegiate boxers: a randomized controlled trial

**DOI:** 10.3389/fphys.2025.1701045

**Published:** 2025-10-02

**Authors:** Yemin Han, Yiqing Xie, Zhen Zhang, Amador García-Ramos

**Affiliations:** ^1^ School of Physical Education, Shanghai University of Sport, Shanghai, China; ^2^ Sport, Exercise and Health Sciences, Loughborough University, Loughborough, United Kingdom; ^3^ School of Athletic Performance, Shanghai University of Sport, Shanghai, China; ^4^ Department of Sports Sciences and Physical Conditioning, Universidad Católica de la Santísima Concepción, Concepción, Chile; ^5^ Department of Physical Education and Sport, Faculty of Sport Sciences, University of Granada, Granada, Spain

**Keywords:** combat sports, strength training, velocity-based resistance training, velocity threshold, lower-limb strength performance

## Abstract

**Background:**

Boxing performance heavily relies on lower-limb strength and power. Velocity-based resistance training (VBT), which adjusts load and repetition volume using real-time velocity feedback, may provide a more individualized and effective approach compared to traditional percentage-based training (PBT). However, its long-term effect on boxing-specific performance outcomes remains underexplored.

**Methods:**

Twenty-eight male collegiate boxers were randomly assigned to a VBT group (n = 14) or a PBT group (n = 14) for an 8-week training program. Both groups performed four sets of each exercise (back squat, Bulgarian split-squat, and deadlift) at 70% of their one-repetition maximum (1RM). The VBT group performed a flexible number of repetitions until their velocity dropped below a 10% threshold, whereas the PBT group consistently performed sets of 5 repetition. Pre- and post-intervention assessments included 1RM strength, countermovement jump (CMJ) height, standing long jump (SLJ) distance, and 30 m sprint run time.

**Results:**

All dependent variables demonstrated significant main effects of “time” (*p* < 0.001; averaged Hedges’ g = 0.44 for VBT group and 0.23 for PBT group). Notably, significant “time” × “group” interactions were observed for the CMJ, SLJ, and 30 m sprint run (*p* ≤ 0.038), whereas no significant interactions were found for 1RM strength measures across exercises (*p* ≥ 0.163). Furthermore, when comparing the magnitude of changes between groups, the VBT group exhibited small effect size improvements in CMJ height (Hedges’ g = 0.41), SLJ distance (Hedges’ g = 0.56), and 30 m sprint time (Hedges’ g = 0.51). In contrast, all other variables only showed trivial (Hedges’ g < 0.20) differences between groups.

**Conclusion:**

Both training programs led to comparable improvements in maximal strength (1RM) across exercises. However, VBT was more effective than PBT in enhancing performance in high-velocity tasks such as vertical and horizontal jumps and sprinting. These findings support the use of VBT to optimize neuromuscular adaptations relevant to explosive actions in male collegiate boxers.

## Introduction

Resistance training (RT), is a form of physical exercise that utilize external loads to exercise muscle groups, enhance neuromuscular fitness and improve athletic performance (Gonzalez-Badillo et al., 2022). Among the various RT variables, training intensity and volume are the two primary determinants of physiological adaptation (Kraemer and Ratamess, 2004; Bird et al., 2005; Scott et al., 2016). Traditionally, RT intensity is prescribed using a fixed percentage of an athlete’s one-repetition maximum (1RM), and the number of repetitions per set is also standardized accordingly (Kraemer and Ratamess, 2004; Scott et al., 2016). Although this percentage-based training (PBT) model is widely used, it has several limitations. For example, assessing 1RM is time-consuming and physically demanding, and it may increase the risk of injury (Ramos, 2024). Moreover, it does not account for daily fluctuations in performance caused by factors such as sleep, fatigue, nutrition, or psychological stress (Gonzalez-Badillo and Sanchez-Medina, 2010). In addition, prescribing a fixed number of repetitions overlooks individual differences in fatigue tolerance, potentially resulting in either insufficient training stimulus or excessive fatigue accumulation (Gonzalez-Badillo and Sanchez-Medina, 2010; González-Badillo et al., 2011).

To address these issues, velocity-based resistance training (VBT) has emerged as an alternative approach that uses movement velocity as a real-time indicator of training intensity and fatigue level (González-Badillo et al., 2011; Orange et al., 2020b; Weakley et al., 2021). Based on the stable relationship between barbell velocity and relative load (%1RM), movement velocity can be used to guide loading prescription (Gonzalez-Badillo and Sanchez-Medina, 2010; Sánchez-Medina et al., 2017). Several studies across diverse populations have shown that lifting with maximal intended velocity produces greater improvements in power- and speed-related variables than deliberately lifting at submaximal velocities (González-Badillo et al., 2014; Pareja-Blanco et al., 2014; Lecce et al., 2025a; Lecce et al., 2025b). Furthermore, VBT introduces a novel method of managing training volume: instead of prescribing a fixed number of repetitions, a set is terminated once a predetermined velocity loss (VL) threshold is reached (Pareja-Blanco et al., 2017a; Pareja-Blanco et al., 2017b). The validity of this method relies on the significant association between the magnitude of velocity loss and multiple markers of mechanical, metabolic and perceptual fatigue (Sánchez-Medina and González-Badillo, 2011; González-Badillo et al., 2017; Rodríguez-Rosell et al., 2020b). This auto-regulatory strategy aims to maintain a high number of repetitions performed at high velocities, manage neuromuscular fatigue, and promote optimal training adaptations (Perez-Castilla et al., 2018; Rodríguez-Rosell et al., 2021).

Recent evidence suggests that different VL thresholds induce distinct neuromuscular and performance adaptations (Pareja-Blanco et al., 2017b; Perez-Castilla et al., 2018; 2020). Higher VL thresholds (20%–40%) tend to promote muscle hypertrophy but are associated with greater fatigue accumulation (Jukic et al., 2023). In contrast, lower VL thresholds (≤20%) are less fatiguing and yield similar or even superior improvements in maximal strength, muscular endurance, and high-speed, short-duration movements such as vertical jumps and sprinting (Pareja-Blanco et al., 2017a; Pareja-Blanco et al., 2020; Rodríguez-Rosell et al., 2020a). Compared to PBT, VBT with repetition volume regulation based on VL has demonstrated superior improvements in explosive performance indicators—such as jump height, sprint time, and maximal strength output—in sports like football, rugby, and track and field (Pareja-Blanco et al., 2017b; Weakley et al., 2020; Yuan et al., 2023). However, this approach remains underexplored in combat sports such as boxing.

Boxing heavily relies on the transfer of lower-limb strength to generate effective punches and footwork (Davis et al., 2013; Chaabène et al., 2015). Given the demands for high-velocity force application and sensitivity to fatigue in training, VBT may be particularly well-suited for boxers (Turner et al., 2011; Loturco et al., 2016). Therefore, this study aimed to compare the effects of an 8-week VBT program using a 10% VL threshold with those of traditional PBT on lower-limb strength performance in male collegiate boxers, providing novel insights into resistance training strategies for boxing. Previous studies indicate that a 6–8 week period is commonly used to capture the initial adaptations elicited by different strength training protocols (Schoenfeld et al., 2017; Pareja Blanco et al., 2020). Based on prior evidence of VBT efficacy across various sports (Banyard et al., 2020; Lecce et al., 2025a; Lecce et al., 2025b), we hypothesized that VBT would lead to greater improvements in jump performance and sprint ability compared to PBT, while no significant differences would be observed in lower-limb maximal dynamic strength (1RM).

## Materials and methods

### Subjects

The sample size for this study was determined using G*Power 3.1 (Faul et al., 2009), with a medium effect size (f = 0.30), α = 0.05, and power (1-β) = 0.80, indicating that at least twenty-four participants were required for repeated measures ANOVA. A total of twenty-eight male collegiate boxers from Shanghai University of Sport (Shanghai, China) voluntarily participated and were randomly assigned to either the VBT group or the PBT group (Table 1). All subjects provided written informed consent after being briefed on the study protocol and potential risks and benefits. To ensure the scientific validity of the data and minimize injury risk, the following inclusion criteria were applied: (1) subjects engaged only in regular technical-tactical training, minimizing other physical exertion; (2) subjects had at least 2 years of RT experience (2-4 sessions per week) (Weakley et al., 2017), and received professional technical evaluation and guidance 2 weeks before testing to ensure proficiency and standardization of exercise techniques, ensuring that performance changes were attributed to the training stimulus and not learning effects; (3) subjects were healthy with no injuries in the 6 months prior to testing. The study was approved by the Ethics Committee of Shanghai University of Sport (Approval No. 102772025RT044).

**TABLE 1:** Baseline characteristics of study participants.

Variable	VBT (n = 14)	PBT (n = 14)
Age (years)	19.6 ± 1.0	19.9 ± 1.0
Height (cm)	181.9 ± 7.4	179.1 ± 7.3
Body mass (kg)	77.9 ± 9.1	78.4 ± 9.5
Boxing experience (years)	6.3 ± 1.4	6.0 ± 1.5

Abbreviations: VBT, velocity-based resistance training; PBT, percentage-based training.

### Experimental design

All tests and interventions were conducted at the Physical Training Research Center of Shanghai University of Sport (Shanghai, China). The baseline testing was conducted over two separate testing days. On the first day, 1RM value tests for various exercises were performed. On the second day, the 30 m sprint run, standing long jump (SLJ), and countermovement jump (CMJ) tests were performed. Participants were asked to avoid staying up late or consuming alcohol prior to testing. The post-intervention testing followed the same procedure as the baseline testing.

### Testing procedures

#### 1RM assessment

This study conducted 1RM assessments for three exercises: back squat (BS), Bulgarian split-squat (BSS), and deadlift. Participants began the 1RM test with an initial load of 20 kg, progressively increasing the load in 10 kg increments until the mean velocity (MV) dropped below 0.5 m·s^-1^. Thereafter, load increments were reduced between 1 and 5 kg to precisely determine the maximal load at which a complete repetition could be performed. Throughout the testing procedure, researchers closely supervised participants to ensure correct technique and safety. For lighter loads (MV > 0.7 m·s^-1^), 3-4 repetitions were performed; for moderate loads (0.5 m·s^-1^ ≤ MV ≤ 0.7 m·s^-1^), 2 repetitions; and for heavier loads (MV < 0.5 m·s^-1^), only 1 repetition was executed. Rest intervals consisted of 10 s between repetitions at the same load and 5 min between different loads. Verbal encouragement and real-time velocity feedback were provided during each repetition to motivate maximal voluntary effort.

For the BS exercise, participants positioned their feet shoulder-width apart or slightly wider, with toes pointing forward. During the descent, knees were allowed to travel slightly beyond the toes until the thighs were parallel to the ground. In the BSS, the rear foot was elevated and placed on a bench while the front foot remained flat on the floor, maintaining an upright torso. The movement initiated from a standing posture, with a controlled descent until the front thigh was parallel to the floor and the front knee approached, but did not contact, the ground. The BSS test was performed on both the dominant and non-dominant legs to assess unilateral strength. For the deadlift, participants stood with feet shoulder-width apart and toes externally rotated by 10°–15°. At the start of the pull phase, elbows were fully extended, gripping the barbell with a mixed grip at shoulder width. The force production sequence began with ground contact through the feet, followed by leg drive and hip extension, lifting the barbell while maintaining an upright posture without excessive forward pelvic tilt. Exhalation was coordinated with the exertion phase.

#### SLJ assessment

Participants adjusted their stance behind the starting line, performed a pre-squat with arm swing, and explosively jumped forward. The distance from the nearest point of contact to the starting line was measured. Participants were not allowed to toe or cross the line during the jump. Participants performed three trials separated by 30 s of rest. The best result was recorded, rounded to two decimal places.

#### 30M sprint run assessment

The 30 m sprint is a commonly used indicator for assessing lower-limb explosiveness, as it not only reflects an athlete’s level of explosive power but also demonstrates the efficiency with which maximal lower-limb strength can be translated into rapid displacement (Vencúrik and Fikar, 2022; Li et al., 2025). After proper warm-up, participants sprinted 30 m from a stationary start, with timing gates at both the start and finish (Smart Speed system, Fusion Sport Inc., Australia). A crouched start was used, and participants sprinted as soon as the “start” command was given. Participants performed two trials separated by at least 3 min of rest. The best time was recorded, rounded to two decimal places.

#### CMJ assessment

CMJ is a widely used test for lower-limb strength performance, utilizing a dual-force plate system (KWYP-FP6035, Kunwei Sport Technology Co., Ltd., Shanghai, China) with a sampling rate of 1,000 Hz. Data were collected using Gameon software (Gameon Sports Science Corp, Shenzhen, China) and analyzed with its integrated software (KW3.1.10.9, Kunwei, Shanghai, China). To reduce the effect of upper-limb swing, participants performed the test with their hands on their hips to avoid torso rotation. Participants performed three trials separated by 30 s of rest. The best result was recorded, rounded to two decimal places.

### Resistance training program

**FIGURE 1:**
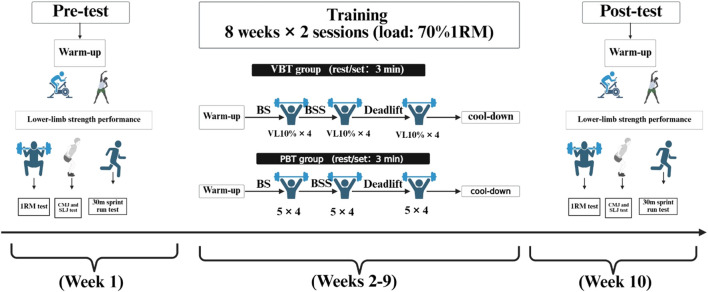
Overview of the experimental design. CMJ, countermovement jump; SLJ, standing long jump; BS, back squat; BSS, Bulgarian split-squat; PBT, percentage-based training; VBT, velocity-based resistance training.

The intervention lasted 8 weeks, with two sessions per week (Figure 1). Both the VBT and PBT groups trained at the same time of the day. After 4 weeks of training, participants’ 1RM for each exercise was retested, and training loads were adjusted accordingly in the PBT group for the remaining 4 weeks. During the lower-limb strength training sessions, the VBT group used a linear position transducer (GymAware Power Tool Version 6.1; Canberra, Australia) to monitor movement velocity. Studies have shown that GymAware demonstrates high reliability across the entire velocity range (Orange et al., 2020a; Mitter et al., 2021). Other training components, such as technical-tactical exercises, remained the same. Before training, all participants performed a warm-up, which included general physical activities as well as boxing-specific preparation drills, lasting 10–15 min, followed by 3–5 min of recovery. During training, the PBT group used a constant load of 70% 1RM, completing 4 sets of 5 repetitions with 3-min rest intervals between sets. The VBT group selected loads based on the velocity corresponding to 70% 1RM (with a target velocity deviation maintained within ±0.03 m/s). Each set was terminated when VL reached the predefined 10% VL threshold. The VBT group completed 4 sets, with 3-min rest intervals between sets. After training, participants performed a cool-down to alleviate exercise-induced fatigue, under the guidance of the same instructors leading the training sessions.

### Statistical analyses

Descriptive statistics are presented as mean ± standard deviation (Mean ± SD). The normality of the data was assessed using Shapiro-Wilk tests, and Levene’s test was used to check for homogeneity of variance. Two-factor mixed analysis of variance test was used to examine the effects of “time” (within-subject factor: pre-test vs. post-test) and “training group” (between-subject factor: VBT vs. PBT) on lower-limb strength performance in boxers. The magnitude of the changes was assessed using Hedges’ g effect size (ES), along with 95%CIs. ES was calculated using pretest SD for within-group and pooled pretest SD for between-group comparisons. ES magnitudes were classified as: trivial (<0.20), small (0.20–0.59), moderate (0.60–1.19), large (1.20–2.00), and extremely large (>2.00) (Hopkins et al., 2009). Statistical significance was set at *p* ≤ 0.05, with all analyses performed using SPSS (version 27, IBM, Armonk, NY, United States).

## Results

**FIGURE 2:**
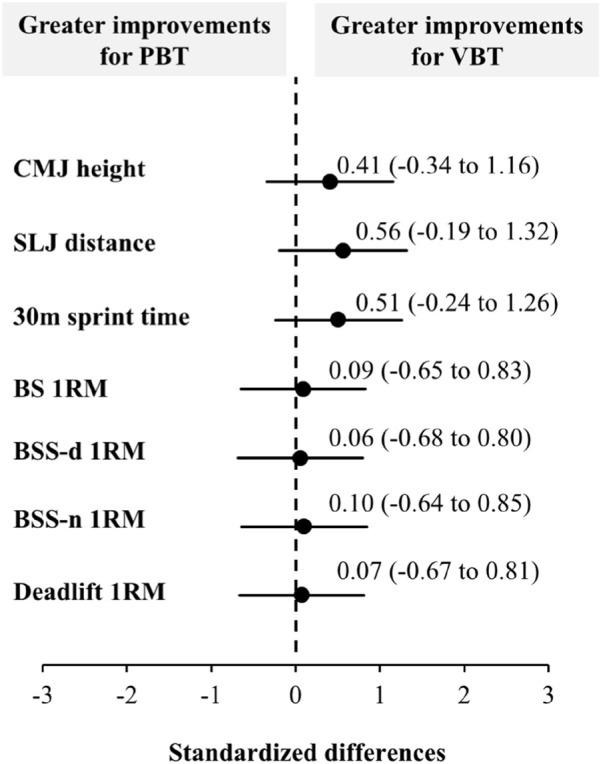
Standardized differences with 95% confidence intervals for the pre to post changes in lower-limb strength performance variables between the VBT and PBT. CMJ, countermovement jump; SLJ, standing long jump; BS, back squat; BSS-d, dominant side in Bulgarian split-squat; BSS-n, nondominant side in Bulgarian split-squat; PBT, percentage-based training; VBT, velocity-based resistance training.

No significant differences were observed between the VBT and PBT groups across any measured variables prior to the intervention (*p* > 0.05). Following the 8-week training program, a significant main effect of “time” was found for all dependent variables (*F* ≥ 27.1, *p* < 0.001) (Table 2). However, the interaction effect between “time” and “group” differed across variables: significant interactions were detected for CMJ, SLJ, and 30 m sprint performance (*F* ≥ 4.8, *p* ≤ 0.038), whereas 1RM strength did not reach statistical significance for any exercise (*F* ≤ 2.1, *p* ≥ 0.163). Furthermore, when comparing the magnitude of changes between groups, the VBT group exhibited greater gains (small ES) in CMJ height (ES = 0.41), SLJ distance (ES = 0.56), and 30 m sprint time (ES = 0.51). In contrast, trivial (ES < 0.20) differences were observed between the PBT and VBT groups for the 1RMs (Figure 2).

**TABLE 2:** Two-way ANOVA comparing the pre to post changes in lower-limb strength performance variables for the PBT and VBT groups**.**

Variable/group	Pre-test Mean (SD)	Post-test Mean (SD)	Hedges g, ES (95% CI)	ANOVA	
				Time	Interaction
Countermovement jump height, cm					
PBT VBT	32.6 (5.40) 33.2 (4.42)	34.0 (5.35) 36.7 (4.48)^a^	0.25 (−0.49, 1.00) 0.76 (0.00, 1.53)	*F* = 27.1 *p* < 0.001	*F* = 4.8 *p* = 0.038
standing long jump, m					
PBT VBT	2.48 (0.18) 2.54 (0.21)	2.52 (0.14) 2.69 (0.17)^a^	0.24 (−0.50, 0.98) 0.76 (−0.01, 1.53)	*F* = 64.7 *p* < 0.001	*F* = 22.5 *p* < 0.001
30 m sprint run, s					
PBT VBT	4.12 (0.29) 4.18 (0.32)	4.10 (0.29) 3.99 (0.30)^a^	−0.09 (−0.81, 0.67) −0.59 (−1.35, 0.16)	*F* = 37.5 *p* < 0.001	*F* = 20.9 *p* < 0.001
Back squat 1RM, kg					
PBT VBT	141.0 (24.6) 135.0 (32.6)	146.9 (21.9)^a^ 143.6 (31.2)^a^	0.25 (−0.50, 0.99) 0.26 (−0.48, 1.01)	*F* = 57.7 *p* < 0.001	*F* = 2.0 *p* = 0.169
BSS-dominant 1RM, kg					
PBT VBT	104.2 (14.0) 96.4 (27.3)	109.8 (15.6)^a^ 103.4 (25.7)^a^	0.37 (−0.38, 1.11) 0.26 (−0.49, 1.00)	*F* = 30.8 *p* < 0.001	*F* = 0.4 *p* = 0.534
BSS-nondominant 1RM, kg					
PBT VBT	96.6 (15.4) 91.4 (26.7)	99.8 (16.0) 97.1 (25.7)^a^	0.20 (−0.54, 0.94) 0.21 (−0.53, 0.95)	*F* = 29.6 *p* < 0.001	*F* = 2.1 *p* = 0.163
Deadlift 1RM, kg					
PBT VBT	154.9 (24.8) 150.4 (29.5)	159.7 (25.2)^a^ 157.1 (29.2)^a^	0.19 (−0.56, 0.93) 0.22 (−0.52, 0.96)	*F* = 33.1 *p* < 0.001	*F* = 1.0 *p* = 0.329

Abbreviations: ANOVA, analysis of variance; BSS, Bulgarian split-squat; PBT, percentage-based training; VBT, velocity-based resistance training; 1RM, one-repetition maximum; ES, effect size = (post-test mean − pre-test mean)/pre-test SD.

^a^ Significant differences compared with pre-test (*p* < 0.05).

## Discussion

This study is the first to systematically compare the differences in strength adaptations between VBT and PBT in boxing, and the main results supported our hypothesis. The findings showed that both training modalities produced similar improvements in maximal strength (1RM). However, compared with the PBT group, the VBT group demonstrated greater enhancements in high-speed action-related measures, such as CMJ height (g = 0.76 vs. 0.25), SLJ distance (g = 0.76 vs. 0.24), and 30 m sprint run time (g = 0.59 vs. 0.09). These differences may be explained by distinct physiological mechanisms: PBT primarily relies on increasing cumulative time under tension (TUT) to promote strength adaptations (Pareja-Blanco et al., 2014), whereas VBT induces neuromuscular adaptations through contraction velocity stimulation (Tøien et al., 2022), such as faster motor unit recruitment, higher firing frequency, and improved intermuscular coordination (Tillin et al., 2011; Del Vecchio et al., 2024).

Consistent with our findings, recent studies using similar velocity-versus-loading designs have also reported comparable improvements in 1RM strength between training modalities, and highlighted contraction velocity as a key determinant of improvements in peak power, rate of force development (RFD), and impulse (Lecce et al., 2025a; Lecce et al., 2025b). Although VBT can enhance high-speed actions, such improvements may be limited by factors such as athletes’ baseline capacities, the short intervention period, and the predominance of neural over structural adaptations. These superior adaptations may be attributed to three key variables that differentiated VBT from PBT: (i) daily-based individualized load adjustment, (ii) flexible volume regulation using VL thresholds, and (iii) continuous real-time feedback on lifting velocity.

Traditionally, direct assessment of 1RM is considered an effective method to evaluate an individual’s maximal strength capacity and subsequently prescribe loads during resistance training programs (Grgic et al., 2020). However, it presents several challenges, including physical, technical, and psychological demands (Ramos, 2024). Moreover, 1RM values are subject to daily fluctuations due to factors such as training fatigue, sleep deprivation, nutritional status, and academic or occupational stress (Gonzalez-Badillo and Sanchez-Medina, 2010; Hirsch and Frost, 2021). To address these issues, individualized load-velocity (L-V) profiles, based on the velocity attained under submaximal loads, have been proposed. These profiles allow for real-time adjustments of training loads, potentially providing more accurate estimations of an athlete’s current 1RM (García-Ramos et al., 2018). This method ensures athletes train at the desired intensity, potentially maximizing performance outcomes while minimizing injury risk (Ramos, 2024).

In addition to load adjustment, VBT regulates training volume by terminating sets once a predetermined VL threshold (e.g., 10%, 20%, or 30%) is reached. Compared to conventional resistance training prescriptions, implementing VL thresholds allows for better fatigue management (Sánchez-Medina and González-Badillo, 2011; González-Badillo et al., 2017), as VL has been shown to correlate strongly with mechanical, metabolic, and perceptual markers of fatigue (Sánchez-Medina and González-Badillo, 2011; González-Badillo et al., 2017; Rodríguez-Rosell et al., 2020c). This autoregulatory strategy helps to maintain high-quality repetitions and tailor training volume to the athlete’s daily readiness (Ramos, 2024), A meta-analysis reported that lower VL thresholds were associated with superior gains in strength and high-speed performance, likely due to the avoidance of excessive neuromuscular fatigue (Jukic et al., 2023).

Another unique feature of VBT is the provision of real-time velocity feedback. Feedback has been shown to significantly enhance acute resistance training performance (Pérez-Castilla et al., 2021). Providing repetition-by-repetition feedback improves athletes’ motivation, concentration, and movement quality, while reducing perceived exertion (Wilson et al., 2018; Weakley et al., 2019). Immediate feedback enables athletes to make rapid adjustments in effort and technique, contributing to a more engaging and effective training environment (Weakley et al., 2023). Studies have demonstrated that consistent feedback not only improves short-term performance but also leads to superior long-term adaptations compared to training without feedback (Letafatkar et al., 2020). For coaches aiming to enhance acceleration and sprint performance, incorporating feedback during resistance training is strongly recommended (Randell et al., 2011).

Despite these promising findings, it must be acknowledged that the VBT intervention simultaneously manipulated three variables: load, volume, and feedback. Therefore, it remains unclear whether the observed superior adaptations were driven by a single factor or the combination of all three. Future studies should aim to isolate these variables to determine their independent contributions to training outcomes.

### Practical applications

Practitioners are encouraged to integrate VBT into strength and conditioning programs for boxing athletes. By employing real-time velocity monitoring to adjust training loads dynamically, VBT can effectively reduce excessive fatigue accumulation while maximizing training efficiency. This is especially advantageous for boxers who must consistently maintain peak physical performance throughout training cycles. Furthermore, the efficiency and safety benefits provided by VBT make it particularly suitable for settings with limited training resources or high training densities. Consequently, athletes can allocate more time to other critical training components, such as skill acquisition and tactical development. Future training program designs should further incorporate individualized VBT protocols to foster sustained development in strength and sport-specific performance among boxers.

## Conclusion

This study is the first to systematically compare the effects of VBT and PBT on lower-limb strength performance in male collegiate boxers. After 8 weeks of training, the VBT group demonstrated significantly greater improvements than the PBT group in CMJ, SLJ, and 30 m sprint performance, whereas both training modalities similarly enhanced 1RM strength. These results confirm the superior efficacy of VBT in enhancing lower-limb dynamic performance compared to conventional PBT methods. Nonetheless, because the VBT protocol combined load, volume, and feedback adjustments, the specific contribution of each factor remains uncertain and warrants further investigation. Finally, we recommend incorporating VBT into boxing training regimens to optimize athletic outcomes.

## Data Availability

The original contributions presented in the study are included in the article/supplementary material, further inquiries can be directed to the corresponding authors.
